# Analysis of Global Gene Expression in *Brachypodium distachyon* Reveals Extensive Network Plasticity in Response to Abiotic Stress

**DOI:** 10.1371/journal.pone.0087499

**Published:** 2014-01-29

**Authors:** Henry D. Priest, Samuel E. Fox, Erik R. Rowley, Jessica R. Murray, Todd P. Michael, Todd C. Mockler

**Affiliations:** 1 Donald Danforth Plant Science Center, Saint Louis, Missouri, United States of America; 2 Division of Biology and Biomedical Sciences, Washington University, Saint Louis, Missouri, United States of America; 3 Department of Botany and Plant Pathology and Center for Genome Research and Biocomputing, Oregon State University, Corvallis, Oregon, United States of America; 4 Waksman Institute and Department of Plant Biology and Pathology, Rutgers University, New Brunswick, New Jersey, United States of America; National Taiwan University, Taiwan

## Abstract

*Brachypodium distachyon* is a close relative of many important cereal crops. Abiotic stress tolerance has a significant impact on productivity of agriculturally important food and feedstock crops. Analysis of the transcriptome of *Brachypodium* after chilling, high-salinity, drought, and heat stresses revealed diverse differential expression of many transcripts. Weighted Gene Co-Expression Network Analysis revealed 22 distinct gene modules with specific profiles of expression under each stress. Promoter analysis implicated short DNA sequences directly upstream of module members in the regulation of 21 of 22 modules. Functional analysis of module members revealed enrichment in functional terms for 10 of 22 network modules. Analysis of condition-specific correlations between differentially expressed gene pairs revealed extensive plasticity in the expression relationships of gene pairs. Photosynthesis, cell cycle, and cell wall expression modules were down-regulated by all abiotic stresses. Modules which were up-regulated by each abiotic stress fell into diverse and unique gene ontology GO categories. This study provides genomics resources and improves our understanding of abiotic stress responses of *Brachypodium*.

## Introduction

Plants are sessile organisms that have evolved an exceptional ability to perceive, respond, and adapt to their environment. Environmental stresses are a major limiting factor in agricultural productivity [1], [2], as plant growth is severely affected by environmental conditions such as cold, high-salinity, drought, and heat [3], [4]. In comparison to *Arabidopsis thaliana* and *Oryza sativa*, relatively little is known about how many agriculturally important cereals (e.g., wheat, corn, barley) respond to abiotic stresses [5]–[8]. The stress-induced transcriptomic responses of plants reveal the molecular mechanisms underlying the abiotic stress response. An understanding of these mechanisms will allow researchers to improve stress tolerance of food crops to enhance agricultural productivity under imperfect growing conditions to ensure the world's long-term food security [9]–[11].

The abiotic stress response occurs in two stages: an initial sensory/activation stage, followed by a physiological stage during which the plant responds to the perceived stress [3], [12], [13]. Once a stress cue is perceived, secondary messengers such as calcium and inositol phosphates [14] and reactive oxygen species (ROS) are produced. The increase in Ca^2+^ is sensed by various calcium-binding proteins that initiate phosphorylation cascades that subsequently activate transcription factors [15], [16]. Transcription factors in turn activate expression of stress responsive genes. This begins the second phase and elicits physiological changes necessary to survive the particular environmental stress (reviewed in [13]). The genes expressed and subsequent physiological changes induced during the second phase are dependent upon the particular abiotic stress encountered. These changes can include modifications to cell membrane components – resulting in changes in membrane fluidity [17], stomatal closure [18], decreased photosynthetic activity [19], [20], and increased production of heat shock proteins (HSPs) or dehydrin cryoprotectants [3].

Previous work in monocot stress responses has been completed in rice (*Oryza sativa* ssp. *japonica* cv. ‘Nipponbare’ and ssp. *indica* cv. ‘Minghui 63’). Expression levels of 20,500 transcriptional units in rice callus treated with abscisic acid (ABA) and gibberellin were evaluated using oligonucleotide arrays [21]. A more comprehensive approach using a microarray querying 36,926 genes was used to profile expression responses of rice to drought and high-salinity stresses in three tissues [6]. Recently, profiling of transcriptional responses to cold stresses in winter barley was performed using a microarray-based approach [22], and the transcriptional responses of three wheat cultivars to cold stress were explored in a separate study using microarray-based approaches [23].

The most significant of the available *Brachypodium* genomics resources is the whole genome shotgun sequence assembly. Illumina sequencing was used to deeply sample a collection of cDNA libraries representing a diverse array of tissues, treatments and developmental stages. [24] These data enabled the efficient identification of transcription units and greatly facilitated the design of a whole genome DNA tiling microarray for *Brachypodium distachyon*, based on the DOE-JGI genome sequence, Illumina- and EST-based empirical transcriptome analysis, and model-based gene predictions. A portion of the array space was used to tile each predicted exon and intron with multiple probes with a predefined resolution. The remainder of the *Brachypodium* genome is tiled with reasonable spacing to cover predicted noncoding/intergenic regions. The *Brachypodium* microarray was designed to have 2,548,624 probes targeting exons and introns, with each gene detected by an average of 60.4 probes and of which 90.9% are targeted by >5 probes (see Methods).

Here, we present a genome-wide survey of *Brachypodium* transcript-level gene expression responses to four abiotic stresses: heat, high salinity, drought, and cold. We found significant differences in responses of the *Brachypodium* transcriptome to the four abiotic stresses in terms of timing and magnitude. We were able to identify 22 modules, 10 of which defined clear biological processes. As expected from studies of other plant model systems, photosynthesis, cell cycle and cell wall expression modules were down-regulated under abiotic stress. We found that the modules up-regulated by salt and drought fell into unique gene ontology (GO) categories, whereas cold stress up-regulated transcription factor (TF) expression, and heat stress increased expression of genes involved in stabilizing protein folding, respectively. The response of *Brachypodium* to heat, high salinity, drought, and cold stress was profiled over twenty-four hours after the onset of stress conditions. This study represents a significant development in genomics resources for *Brachypodium*, a close relative of many agriculturally and economically important cereal crop species.

## Results

### Overall Differential Expression Analysis

Drought, high-salinity, cold, and heat are four important abiotic stresses that adversely affect the productivity of plants. We surveyed *Brachypodium* transcript-level gene expression responses to these stresses using the Affymetrix *Brachypodium* Genome Array (BradiAR1b520742). This microarray queries all annotated genes in the *Brachypodium* genome with multiple individual probes targeting each gene. The response of *Brachypodium* to heat, high salinity, drought, and cold stress was profiled in an asymmetric time-course over the twenty-four hours immediately following onset of stress conditions. This allowed us to monitor the transcriptional responses of the plant to stress rather than endogenous circadian or diurnal rhythms. Biological triplicate samples were taken from control and stressed plants at each time point.

Overall, 3,105 genes were significantly up-regulated and 6,763 genes were significantly down-regulated in response to at least one abiotic stress. In response to cold, heat, salt, and drought stresses 40, 1,621, 1,137, and 5,790 genes were significantly down-regulated, respectively. In contrast, 447, 458, 1,565, and 2,290 genes were significantly up-regulated in response to cold, heat, salt, and drought stress, respectively.

**Figure 1:**
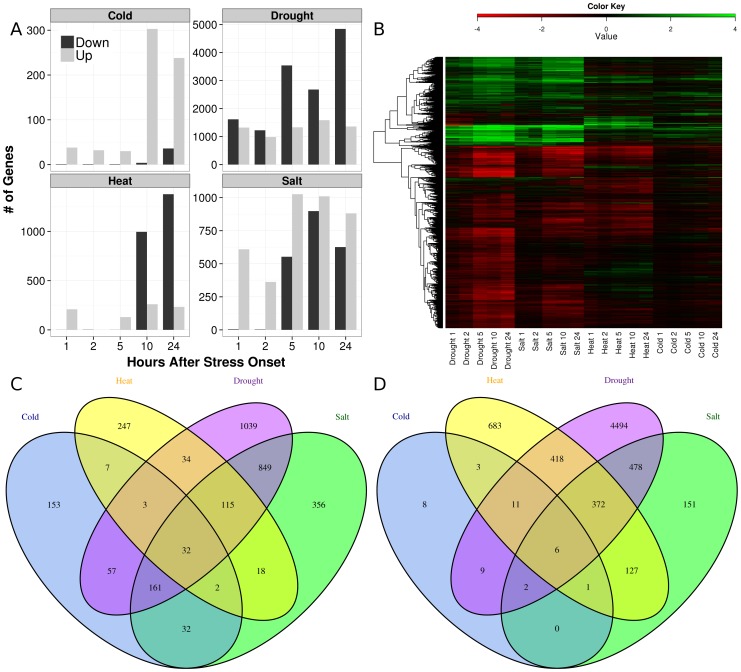
Differential expression of *Brachypodium distachyon* genes in response to stress. **A**. Numbers of genes up-regulated (light grey bars) and down-regulated (dark grey) are shown as a function of time in hours after stress onset. **B**. Heatmap of expression differences between control and indicated stress arrays. Similar expression profiles are clustered in the dendrogram. Positive (green) and negative (red) differences between stress and control arrays are shown for all genes called as differentially expressed by SAM analysis. Columns are time points. Expression values are saturated at +/− 4 RMA, for display purposes. **C.** Venn diagram showing overlap of up-regulated genes in response to the four assayed abiotic stresses: cold (blue), heat (yellow), drought (purple) and salt (green). Area of overlaps is not proportional to the overlap. The numbers of genes in each region of the diagram are indicated. **D.** Venn diagram depicting intersections of sets of down-regulated genes in response to the four assayed abiotic stresses.

The overall number of genes differentially expressed in each stress condition increased over time (**Figure 1A**); the directionality of differential expression differed strikingly with the type of stress. The cold stress response consisted almost entirely of up-regulated genes; very few genes were down-regulated at twenty-four hours (**Figure 1A**, top left). In contrast, the response to heat stress was primarily down-regulation (**Figure 1A**, bottom left). Up-regulation of certain genes in response to heat stress response was observed after 1 hour, but no significant differential expression was observed at 2 hours after onset of stress. After 10 and 24 hours of heat treatment, more than 1,000 genes were down-regulated. Between 1,000 and 2,000 genes were up-regulated at all time points of drought treatment (**Figure 1A**, top right). Down-regulation of genes was low in the early phases of drought response and increased drastically as the treatment continued beyond 2 hours. More than 2,500 genes were differentially expressed 5, 10, and 24 hours after drought onset. Early in the response to salt stress, only up-regulation of genes was observed. At 5 hours post-onset, down-regulation was observed in conjunction with up-regulation with neither as dominant as was seen in the other three stresses (**Figure 1A**, bottom right).

Drought and salt stresses yielded the most similar patterns of variance, whereas the cold and heat stress responses differed strongly from each of the other two stresses and from each other. Similarities were observed in the heatmap depicting hierarchical clustering of the expression data (**Figure 1B**) in which the Robust Multi-array Average (RMA) [25] expression value differences between mRNA abundances in control and stress-treated plants are plotted for all stress conditions. The overall similarity between the salt and drought stress responses can also be seen in this heatmap and is also reflected in the principal component analysis (PCA) results (**Figure S1**).

A large number of genes are differentially expressed only under drought stress (purple ovals, **Figures 1C and 1D**). In response to drought treatment, 1,039 genes were up-regulated and 4,494 were down-regulated. Only about half of the genes differentially expressed in the heat treatment were also responsive to drought (1,088 of 2,079 genes responsive to heat were also responsive to drought). Further, 44.7% of all genes differentially expressed in response to heat stress were unique to that response (930 of 2,079, compare yellow to purple ovals in **Figures 1C and 1D**). Only about 25% of genes differentially expressed upon salt treatment were independent of the drought response (687 of 2,702), and even fewer were unique to salt (507 of 2,702, 18.8%; compare green to purple ovals in **Figures 1C and 1D**). The response to extended cold treatment had strong overlap with the drought response as well. Only 206 genes were responsive to cold stress and not to drought treatment (206 of 487, 42.3%), and 161 genes (of 487 differentially regulated by cold relative to unstressed plants) were uniquely regulated by cold stress (compare blue to purple ovals, **Figures 1C and 1D**). From these analyses, the complex nature of the timing of gene regulation in response to stresses (**Figure 1A**), the differences in intensities of differential expression in response to stresses (**Figure 1B**), and the extensive overlap among genes regulated during stress responses (**Figures 1C and 1D**) are apparent.

### Network Analysis of Stress Response in *Brachypodium*

**Figure 2:**
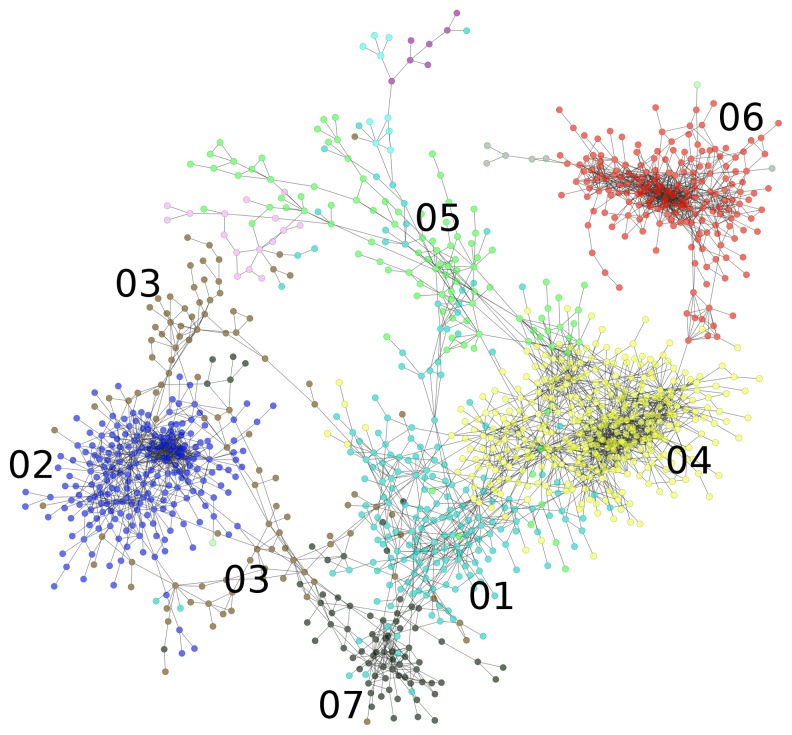
Weighted gene co-expression network of *Brachypodium* stress responsive genes. Major network modules are labeled by proximal numbers, which are identical to those listed in **Tables 1**, **2**, and **3**. Tight node grouping indicates mutually strong edges and therefore high adjacency. All adjacency values plotted are greater than 0.45.

In order to further analyze the systematic transcriptional responses of *Brachypodium* to abiotic stresses, we performed weighted gene co-expression network analysis (WGCNA) on data collected on the 9,496 differentially expressed genes using the WGCNA package in R [26]. Gene modules are composed of genes that share similar profiles and have high correlations with each other. The weighted interaction network is shown in **Figure 2**. Nodes (genes) are connected by edges (co-expression relationships). The connection between two nodes was determined by the correlation between the expression levels of the genes those nodes represent across all experiments used in the analysis.

**Figure 3:**
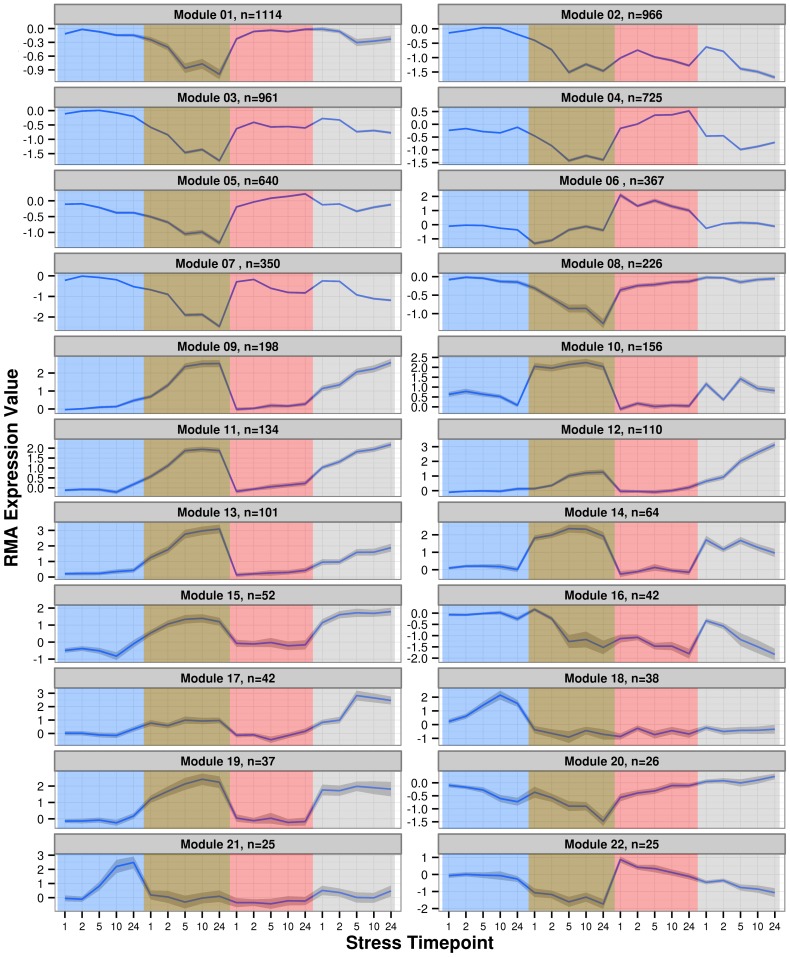
Expression profiles of modules as a function of time in each stress condition. Shaded area around lines indicates standard error. Values plotted are the average point-by-point RMA expression value differences between control and stress arrays for the member genes of the module. N indicates the cardinality of the module in question. Color overlays indicate stress, from left to right: cold (blue), drought (brown), heat (red), and salt (grey).

This analysis resulted in a network that grouped 6,399 genes into 22 modules, the most strongly interconnected of which are shown in Figure 2. The expression profile of each module is shown in Figure 3 as the average difference in RMA expression level between treatment and control arrays. The modular response of *Brachypodium* to abiotic stress was dominated by expression changes in response to the drought stress (**Figure 3**). Differential expression of modules in response to stress was determined by a requirement that an average expression profile must differ from that of the control by one RMA-normalized expression value at one time point under the given stress. Using this criterion, only one module was not responsive to drought stress (module 21; **Figure 3**, lower left). Nineteen of the 22 modules were either stress-specific in their response or responded to only one other stress in addition to drought stress. The remaining three modules are module 16, module 02, and module 07, which were all down-regulated in response to heat, high salinity, and drought stresses. No module was responsive to all four abiotic stresses. Lists of genes in each of the 22 modules may be found in **File S1**.

### Functional Annotation and Promoter Analysis

The combination of the functional annotations of the genes that comprise these modules with their expression profiles shed light on how the plant responds to abiotic stress conditions. Co-regulation is undoubtedly achieved through a combination of transcriptional and post-transcriptional regulation. The grouping of genes facilitated direct analysis of promoters to identify condition-specific over-represented *cis*-regulatory DNA elements. To assign functions to the modules, the module gene lists were analyzed using AgriGO (http://bioinfo.cau.edu.cn/agriGO/analysis.php) [27]. We also analyzed 500 nucleotides from the promoter regions of each of the genes in each module using the Element software package to identify over-represented DNA elements [28].

The module-wise enrichment of GO terms and DNA sequences contained in promoters is shown in **Table 1**. There was a moderate correlation between the number of genes in the module with both the number of GO terms and with the number of DNA sequence elements found to be enriched within that module (Pearson's r: 0.616 and 0.755, respectively). This general correlation between module size and enrichment discovery is expected; however, there were exceptions to this general trend. For example, module 05 is ranked fifth in module size, with 640 member genes, but was not enriched for any GO terms (**Table 1**), although most (585) genes were associated with at least one GO term. Eleven modules were not enriched for any GO terms, and twelve were not uniquely enriched for any GO terms. The modules with no GO-term enrichment varied in size from the minimum size (N = 25) to 640 members (module 05) (**Table 1**, column ‘N’). Upon examination of the GO-terms enriched in each particular module, a pattern of enrichment was often apparent. A selection of the GO-terms enriched in each module, along with the relevant statistics, is shown in **Table 2**. AgriGO output for all 22 modules may be found in **File S2**.

**Table 1:** Module membership and functional and regulatory enrichment.

Module	N	Undefined Genes	Unique GO terms	Total GO terms	Unique DNA Elements	Total DNA Elements
Module 01	1114	96	20	81	60	235
Module 02	966	70	59	75	299	441
Module 03	961	74	27	53	56	208
Module 04	725	39	55	101	323	504
Module 05	640	52	0	0	90	225
Module 06	367	18	11	13	107	151
Module 07	350	18	54	110	97	145
Module 08	226	22	0	0	5	24
Module 09	198	15	1	7	12	45
Module 10	156	6	0	15	190	354
Module 11	134	5	3	4	0	8
Module 12	110	2	0	0	32	69
Module 13	101	7	0	0	8	50
Module 14	64	4	0	0	9	37
Module 15	52	0	0	0	4	12
Module 16	42	1	1	2	3	17
Module 17	42	0	0	0	8	13
Module 18	38	2	4	25	1	15
Module 19	37	3	0	0	1	26
Module 20	26	4	0	0	0	0
Module 21	25	2	0	0	6	12
Module 22	25	1	0	0	1	1

**Table 2:** Specific GO terms uniquely enriched in a selection of network modules.

Module	GO-term	Description	FDR
Module 01	GO:0004812	aminoacyl-tRNA synthetase activity	1.90E-05
	GO:0006418	tRNA aminoacylation for protein translation	8.80E-06
	GO:0006800	oxygen and reactive oxygen species metabolic process	0.022
	GO:0005525	GTP binding	0.039
	GO:0016875	ligase activity, forming carbon-oxygen bonds	1.90E-05
Module 02	GO:0007049	cell cycle	0.0059
	GO:0006260	DNA replication	3.30E-5
	GO:0034728	nucleosome organization	0.00045
	GO:0009832	plant-type cell wall biogenesis	0.00063
	GO:0000271	polysaccharide biosynthetic process	0.016
Module 04	GO:0003899	DNA-directed RNA polymerase activity	7.8E-07
	GO:0006281	DNA repair	0.00082
	GO:0033279	Ribosomal subunit	3.40E-13
	GO:0006364	rRNA processing	1.60E-9
	GO:0008026	ATP-dependent helicase activity	0.00091
Module 06	GO:0031072	heat shock protein binding	0.0012
	GO:0006457	protein folding	2.00E-21
	GO:0009408	response to heat	4.40E-19
	GO:0050896	response to stimulus	4.70E-04
	GO:0010035	response to inorganic substance	0.0043
Module 07	GO:0015979	Photosynthesis	3.20E-45
	GO:0033014	Tetrapyrrole biosynthetic process	1.9E-10
	GO:0006091	generation of precursor metabolites and energy	2.60E-21
	GO:0009765	photosynthesis, light harvesting	2.90E-18
	GO:0010114	response to red light	1.9E-06
Module 09	GO:0009415	response to water	0.0094
Module 11	GO:0009072	aromatic amino acid family metabolic process	0.0062
	GO:0022804	active transmembrane transporter activity	0.038
Module 18	GO:0006351	transcription, DNA-dependent	0.0018
	GO:0016070	RNA metabolic process	0.0076
	GO:0065007	biological regulation	0.0084
Module 16	GO:0016740	transferase activity	0.0088

Even in small modules with the minimum number of genes and no GO-term enrichment, we found over-representation of certain DNA sequences in member gene promoter sequences. Only module 20 was not enriched for any GO terms and had no over-represented DNA elements (**Table 1**). The over-representation of short regions of DNA sequence in the promoters of module member genes may provide insight into the transcriptional circuitry that mediates the regulation of the module. Twenty-one modules had at least one significantly over-represented DNA element (FDR-corrected p-value<0.01). Only two modules had no unique significantly over-represented DNA elements (**Table 1**, modules 11 and 20). Nine of the 22 modules had at least 32 unique elements over-represented in the promoters of their member genes (**Table 1**, column ‘Unique DNA Elements’). Especially in conjunction with the functional annotation of modules via GO-term enrichment, the specific DNA elements which were uniquely enriched show how the transcriptomic responses of *Brachypodium* to abiotic stress compare to other plant systems (**Table 3**). In total, 1,312 elements of 5 to 8 nucleotides long were uniquely associated with specific modules (**File S3**). Element output pertaining to significant DNA motifs can be found in **File S3**.

**Table 3:** Specific short DNA sequences found to be statistically enriched in the promoters of module member genes.

Module	DNA Element	Number of Hits	Number of Promoters	FDR
Module 01	TTAAAAA	346	267	4.94E-08
	TTTAAAA	301	197	1.71E-07
	CTCGTC	423	342	3.52E-05
	ACGTGGGC	139	120	6.03E-05
	CGGCC	380	299	4.80E-05
Module 02	CAACGGTC	57	48	3.79E-17
	AACGGCT	90	79	1.02E-09
	AGCCGTTG	47	39	2.43E-09
	CCAACGG	121	104	2.43E-08
	CAACGGC	115	98	5.38E-05
Module 04	AAACCCT	311	248	2.02E-69
	AGCCCAA	161	134	1.86E-14
	AGGCCCA	211	169	1.02E-28
	AAGCCCAT	57	50	2.57E-11
	GCCCAAC	115	100	1.86E-08
Module 05	ACAAAA	550	345	2.00E-05
	CAATA	617	368	7.05E-08
	ACAATA	197	151	4.04E-05
	ACAATAA	80	71	6.02E-06
	AATAA	1078	463	1.71E-05
Module 06	GAACCTTC	33	30	3.47E-15
	CTAGAAG	55	46	9.78E-11
	CTTCCAGA	28	26	3.98E-10
	AAGCTTC	61	40	1.01E-07
	GAAGCTTC	20	20	1.04E-06
Module 07	ACGTGGC	69	55	4.83E-12
	CCACGTC	59	53	1.39E-07
	GACGTGGC	25	21	5.88E-06
	CACGTGGC	26	20	1.27E-06
	CCTATC	92	81	1.12E-09
	GGGATA	83	78	7.11E-07
	AGATAA	126	105	0.00026
Module 09	ACGTAT	50	32	3.91E-05
	ACGTATA	23	14	1.14E-05
	ACACGTA	31	28	1.38E-06
	CACGTAC	36	28	1.29E-05
	CGTAA	118	83	0.000276
Module 10	CGATCG	47	35	0.00227
	CCGATCG	28	18	0.00049
	ATCGC	122	83	0.00424
Module 12	GTACGTA	27	13	6.08E-06
	GTACAC	41	36	1.44E-05
	ACGTACG	27	14	2.08E-05

### Undefined Module Members

The lists of genes in modules were searched for genes which were identified as lacking useful descriptive annotations or as encoding proteins of unknown function. In all, 3,492 of 26,552 genes in the *Brachypodium* annotation version 1.2 were identified as lacking functional descriptions. In addition to those genes which are of interest due to the combination of their functional annotation and expression profile, genes without functional descriptions can be implicated in specific roles in abiotic stress, even if their function is unknown. The population of genes which are both undefined and members of modules are shown in **Table 1**.

### Network Plasticity

Plasticity of gene regulatory circuits is an expected property of biological systems. There are multiple methods by which the expression relationship between a regulator gene and a target gene may change in response to varying conditions. The regulatory relationship between such gene pairs may change as a result of chromatin rearrangement or DNA methylation [29], [30], both of which have been shown to be responsive to stress in plant species [31], [32]. It is also conceivable that the abundance of the mRNA encoding a particular regulator could be detached from the target expression levels by protein modifications that alter either the activity or degradation rate of the protein in question [33], [34]. The expectation that a transcription factor and target gene pair which interacts will generate correlated expression measurements may not reflect biological reality in all cases.

**Figure 4:**
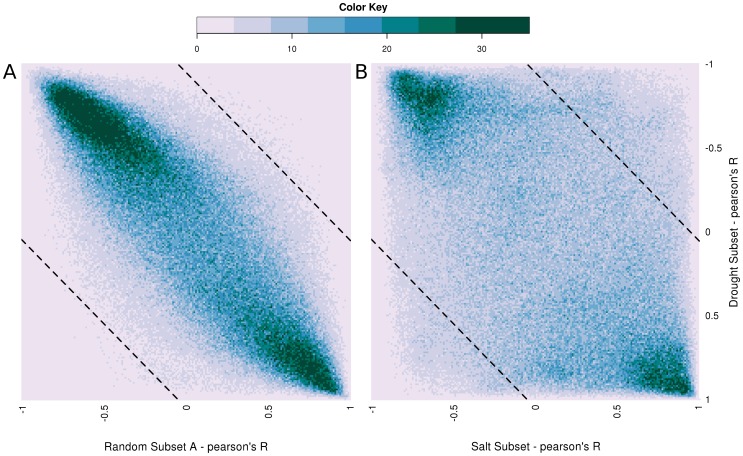
Scatterplot of transcription factor/target gene correlations. The x- and y-coordinates of any single pair of genes is determined by their correlation in the indicated subset. Colors are determined by the number of pairs that fell at a particular point according to the scale shown. Dashed lines indicate the minimum difference required before a TF-TG pair's correlations were considered significantly different between conditions. **A.** The correlations of TF-TG pairs in a random subset of data are compared against the correlations of those pairs in the drought assays. **B.** The correlations of TF-TG pairs in the salt stress and drought stress datasets are plotted. Large amounts of scatter are observed, in contrast to limited scatter in random samples, indicating that when compared across conditions, TF-TG correlations can be highly plastic.

**Figure 4** shows heatmap-scatterplots of transcription factor/target gene (TF-TG) pairs in correlation space. TF-TG pairs are plotted according to their pairwise correlations in each of the shown conditions. Transcription factor/target gene pairs are defined as all possible pairings of genes differentially expressed in the two conditions of interest. Transcription factors are defined via a combination of sequence homology and InterProScan results (see Methods) [35]. The x-coordinate of a TF-TG pair is determined by the pairwise Pearson's correlation between that TF-TG pair in the indicated subset of stress data. The y-coordinate of that TF-TG pair is determined by the pairwise Pearson's correlation of that pair in the subset of stress data drawn from the drought experiment. The heatmap value is determined by the total number of TF-TG pairings with any particular combination of correlations. **Figure 4A** shows the distribution of pairwise TF-TG correlation changes between a random subset of the stress data and the subset of data drawn from the drought experiment, as an indication of what would be expected based on random changes of expression patterns. **Figure 4B** shows the distribution of pairwise TF-TG correlation changes between salt and drought stress data subsets.

In the salt-drought comparison, 146 TFs and 1910 non-TF genes were differentially expressed under both stress conditions. Based on the calculated threshold of Δr = 0.97 for the salt and drought comparison (see Methods), 27,916 of 276,950 TF-TG pairings (10.1%, **Table 4**) showed significant differential correlation across conditions, indicating possible plasticity in the relationship between the TF and TG of the pair (**Figure 4B**, top right and bottom left). The remaining 249,034 gene pairings showed less than significant changes in correlation across conditions. **Figure 4A** shows a representative distribution of correlation changes between gene pairs populated by a random permutation of the same data underlying **Figure 4B**. In distributions created by random permutation, an average of 1368.1 gene pairs per permutation were found to have significant changes in correlation based on the threshold of Δr = 0.97 for the same salt-drought comparison, corresponding to the targeted maximum FDR of 0.05 or less (**Table 4**). In all pairwise stress condition comparisons, between 0.9% and 24.9% of gene pairings were found to have potentially plastic relationships (salt/heat and salt/cold, respectively, **Table 4**).

**Table 4:** Putative network plasticity present between all pairwise conditional comparisons.

Stress A	Stress B	Gene Pairings	Plastic Pairs	Average False Positives	FDR	Δr cutoff
Drought	Salt	276,950	27,916 (10.1%)	1368.1	0.049	0.97
Drought	Cold	16,665	2,921 (17.5%)	144.9	0.049	0.96
Drought	Heat	70,434	4,890 (6.9%)	239.9	0.049	0.98
Salt	Heat	26,562	241 (0.9%)	11.9	0.049	1.35
Salt	Cold	8,132	2,027 (24.9%)	94.8	0.047	0.94
Heat	Cold	522	128 (24.5%)	6.0	0.047	0.88

### Stress Responsive Modules in *Brachypodium* Transcriptional Circuitry

The motivations behind linking groups of genes to specific expression profiles in response to stress are multifold. First, modules represent regulatory relationships, indicating how *Brachypodium* reacts in a transcriptional and post-transcriptional manner to abiotic stresses. Second, the expression profiles themselves allow interrogation of the transcriptional regulatory circuitry that allows *Brachypodium* to achieve steady-state levels of stress-responsive transcripts at the appropriate time. This provides links between specific sequences present in the upstream regions of genes, key regulators (e.g. transcription factors), and traits of agricultural and economic interest.

Of all differentially expressed genes, 3,097 (32.6%) were not associated with a module. Different applications of stress, stress treatment severity, temporal distribution of sampling, and temporal density of sampling may enable association of many of these genes with these or other modules to more completely describe the stress response system of *Brachypodium*. Here, four abiotic stress treatments were used: heat, drought, high-salinity, and cold. We did not examine abiotic stresses such as high intensity light, UV, or chemical inducers of reactive oxygen species (ROS). With data on additional stresses, we will be able to associate more genes with over-arching modes of stress response.

### Conserved Abiotic Stress Responses

#### Photosynthesis

Several sub-systems in plants are affected by multiple stresses. Photosynthetic activity (either capacity or efficiency) is known to be down-regulated or depressed upon heat stress [36], drought stress [37], salt stress [20], and cold stress [19]. One of the modules we identified, module 07 (**Figure 3**, top left), is comprised of 350 genes that are very strongly enriched for genes annotated with GO-categories related to photosynthesis, chlorophyll biosynthesis, light response and harvesting, and the chloroplast (**Table 2**, **File S2**). For example, of the 143 genes in *Brachypodium* annotated with GO:0015979 ‘Photosynthesis’, 50 are present in this module (a significant enrichment with FDR-corrected p-value of 3.2×10^−45^). This module was down-regulated in drought, heat, and salt stresses (**Figure 3**). This indicates that under abiotic stress *Brachypodium* down-regulates photosynthesis as observed in several other plant systems [19], [20], [36], [37]. As these genes associated with photosynthesis are affected by several stresses in a coordinated manner, these stresses likely modulate a common transcriptional circuit.

Eight genes in module 07 were found to lack functional descriptions (see Methods) – these loci were investigated further using the comprehensive Phytozome database (phytozome.net) [38]. This search revealed that these loci do not have functional annotations in *Brachypodium*, nor do their best homologs in other monocot species have functional annotations either. The co-expression of these genes with the other genes in module 07 indicates that they likely have some role in mediating either photosynthesis, or the regulatory response of photosynthesis-related genes to abiotic stresses in *Brachypodium*. The function of each of these loci must be elucidated by molecular and genetic analysis.

The ABRE (ACGT-containing abscisic acid response element) is a known *cis*-regulatory motif in *Arabidopsis thaliana* that contains an ACGT core and is responsive to drought [39]. This sequence was found in the promoter regions of many genes in the photosynthesis module (module 07), the water-response module (module 09, **Table 2**) and a transcription factor enriched module (module 10, **Table 2**, **File S2**). Notably, even though the photosynthesis module and the signaling module (module 03) share highly similar expression profiles, this core sequence was not significantly enriched in the promoters of genes in the signaling module. The photosynthesis module is down-regulated under drought stress, whereas modules 09 and 10 are up-regulated under the same stress (**Figure 3**). Thirteen variations of the ABRE (including the ACGT core with differing flanking regions) were found in the photosynthesis module (**Table 3**, **File S2**). Negative regulation of the photosynthesis module by the ABRE in response to drought stress was expected based on previous studies [40]–[42]. Forms of the ABRE were also over-represented in the promoters of genes in modules 11, 12, 13, 14, 15, and 19. These modules were not found to be over-represented for any GO-terms. However, these modules were up-regulated by both salt and drought stresses. The functional roles of these modules remain to be explored.

The photosynthesis module (**Figure 2** **,** **Table 2**) is strongly enriched for genes related to photosynthesis and was severely down-regulated in drought and moderately down-regulated in heat and salt stresses. These genes were not down-regulated in cold stress, but the overall depression of photosynthesis-related genes appears to be conserved in *Brachypodium* (**Figure 3**, top left). The relative severity of the stress conditions applied no doubt plays a role in the relative levels of regulation observed for this module.

#### Plant growth

Plant growth is severely affected by environmental conditions such as cold, high-salinity, drought, and heat [3], [4]. Module 02 (**Figure 2**) is characterized by an expression profile similar to the photosynthesis module (module 07), though it shows larger negative changes in expression under both salt and heat stress treatments. Module 02 is enriched for genes annotated with GO-terms related to DNA replication, chromatin and nucleosome assembly, the cell cycle, and cell wall biogenesis (**Table 2**, **File S2**). The down-regulation of these genes suggests that an early response of *Brachypodium* to abiotic stresses is to suppress cell growth, DNA replication, and the cell cycle.

Similar to those genes in module 07, no functional annotation could be attributed to 77 loci in module 02, though they are differentially expressed in response to abiotic stress, and co-express with the rest of the genes of module 02. Given that these genes are co-expressed with the rest of the genes in module 02, it is likely that they play some role in the functions that are associated with their module, such as the cell cycle, DNA replication, or cell wall biogenesis. The specific functions of each of these genes must be described in follow-up molecular and genetic experiments.

The Mitosis-Specific Activator (MSA) motif includes the core sequence ‘AACGG’ and is associated with G2/mitosis specific genes in *Arabidopsis* [43]. At*MYB3R4* has been shown to directly bind to this motif *in vitro* [43]. Module 02 is enriched for GO categories related to DNA replication, microtubule-based processes, chromatin, and nucleosome assembly. Thus, the ‘cell-cycle’ module is down-regulated under stress, indicating a suppression of these systems, which may result in a lengthened G2 phase and a slowed cell cycle. The promoters of the cell cycle module are heavily enriched with the ‘AACGG’ core of the MSA motif, as well as its reverse complement (**Table 3**). Notably, the sequence ‘AACGG’ was found 907 times in 540 of the 966 gene promoters in this module (FDR-corrected p-value = 0.00043). Six distinct 8-nucleotide sequences containing this core were found 275 times (all six with FDR-corrected p-value<3.94×10^−5^, **Table 3**). This core was also enriched in module 10; we observed this sequence 168 times in 95 of the 156 promoters (FDR-corrected p-value = 0.001, **File S3**). Small plant stature and decreased yield are a major consequence of abiotic stress in plants[3], [4] [41]. A decrease in expression of genes activated by the MSA motif could conceivably result in a much slower or completely suspended cell cycle in the G2 phase. *Arabidopsis* plants deficient in TFs associated with the MSA showed pleiotropic dwarfism and other developmental and growth defects [43]. The putative ortholog of At*MYB3R4*, *Bradi2g31887*, is a member of the signaling module (module 03). The signaling module is also enriched for microtubule related GO-terms, as well as many signaling-related GO-terms. However, none of the unique significantly enriched DNA sequence elements present in the promoters of module 03 contain the MSA core nor is the MSA core itself enriched in gene promoters from this module (**Table 3** **, File S3**). Elucidation of the relationship between the MSA and TFs such as that encoded by *Bradi2g31887* that may bind the MSA and suppression of the cell cycle by down-regulation of MSA-controlled genes will require further study.

#### Calcium-mediated stress response

Calcium receptors and calcium binding proteins are important components of plant abiotic stress response. Calcium levels increase early in the cellular response to cold stress [44], and a link exists between calcium binding proteins and the cold-response CBF pathway in *Arabidopsis*. A model was recently proposed linking an increase in cellular Ca^2+^ levels with positive transcriptional control of CBF/DREB loci in *Arabidopsi*s [16]. Calcium levels also play a key role in drought and salt stress responses. At*CBL1* is an *Arabidopsis* calcium sensor that is up-regulated in response to salt, drought, and cold stresses [45]. Evidence suggests that calcium sensing plays a role in heat stress response in monocot species as well [46]–[48].

Using homology to other model systems combined with annotation via InterProScan, 359 genes were associated with GO:0005509 (‘calcium ion binding’) or were associated with the phrase ‘calcium binding’. Expression data for these genes was hierarchically clustered and plotted in a heatmap (**Figure S2**) that shows the expression of calcium ion binding genes in *Brachypodium* in response to the four assayed stresses. The expression levels of calcium ion binding loci were strongly affected by abiotic stress and were highly-correlated in drought and salt responses, although were independent in heat and cold stress responses. Principal component analysis of the expression data of the 359 genes annotated with GO:0005509 (**Figure S3**) revealed that trends in expression of the 359 genes were highly similar to the trends in expression of differentially expressed genes overall. The first principal component was the strongest factor in later hours of drought and salt stress and explained 65.44% of the total variance of the expression data associated with the 359 putative calcium ion binding loci.

Of the 359 putative calcium ion binding loci, 88 genes were part of a module. This is significantly fewer than would be expected by chance alone (average expected overlap: 242 genes, Z-score −18.1). Sixteen of the 22 modules contained at least one putative calcium-binding locus. No module was enriched for GO:0005509 (‘calcium ion binding’). The large distribution of calcium responses to abiotic stress (**Figure S2**) indicate that there are multiple regulatory pathways that trigger calcium ion binding protein expression and that these loci play a role in mediating the response of *Brachypodium* to the four assayed stresses. Further, their significant under-representation among modular loci suggests that the response of individual differentially expressed calcium loci does not conform to the major modes of stress response. The regulatory circuits that control calcium ion binding loci appear to be specific to these individual genes. Prior studies provided evidence that calcium ion levels, calcium ion binding protein levels, and abiotic stress responses are linked in multiple plant systems [16], [45], [48]. Our analysis confirms that calcium ion sensing and calcium ion binding loci are responsive to abiotic stress in *Brachypodium*. We found no evidence of a centralized calcium response system.

#### Novel and uncharacterized modules

Module 05 is down-regulated under drought stress but not differentially expressed under any of the other three stresses. Module 05 was not enriched for any GO terms (**Table 1**). Of the 640 genes in the module, 585 genes were annotated with at least one GO-term. The promoter regions of the genes in this module were enriched for 225 specific conserved motifs; of these, 90 are uniquely enriched in module 05 (**Table 1**). These include the core CAATA (FDR-corrected p-value 7.05×10^−8^) and the variant ACAAAA (FDR-corrected p-value 2×10^−5^). The PlantCARE [49] database lists the core CAATA as part of an Auxin Response Element (ARE) in *Glycine max*.

Like module 05, module 08 is down-regulated only in drought. This module has 226 member genes and is not enriched for any GO terms. Twenty-four DNA sequence motifs were significantly enriched in promoters of module 08. Uniquely significant motifs included TCCTTCA, CCCGAC, and CCGAAA. These motifs are similar to the CRT/DRE DNA TF-binding site, RCCGAC [50], [51]. Conserved *cis-*acting elements similar to those found in the promoters of modules 05 and 08 have been observed in other species, lending weight to the hypothesis that these DNA sequences could be responsible for driving the module-wise expression profiles observed here. No enriched functional terms could be associated with modules 05 and 08. An extended examination of gene expression responses to abiotic stress – especially stretching into the days after stress onset – may reveal the functional roles these modules play.

## Discussion

This study provides insight into the regulatory responses of *Brachypodium* to four abiotic stresses. Application of the *Brachypodium* genome-scanning tiling array resulted in deep profiling of the transcriptional response to abiotic stress. The data and analysis provided here will be an excellent resource for researchers utilizing *Brachypodium* as a model system, as will the web-based resources provided for community use.

### Conserved Modular Responses

Previous studies in rice observed a high overlap between gene sets differentially expressed in response to drought and high salinity stresses [6]. Our work captures a similar response in *Brachypodium*, with roughly 75% of the genes differentially expressed in response to salt also differentially expressed in response to drought. Similarities in overall pattern and variance of the responses to drought and high-salinity are also seen in the Principal Component Analysis (PCA).

Many systematic responses to abiotic stress in *Brachypodium* could be characterized on the modular level – these responses are coordinated in independent stresses. This is reflected in the very strong enrichment of photosynthesis-related genes in module 07 (**Table 2**), and the expression pattern of the same module in response to drought, heat, and high-salinity stress (**Figure 3**). The well-characterized behavior of photosynthesis systems in response to stresses [19], [20], [36], [37], combined with the distinct co-expression profile of module 07 lends further weight to the hypothesis that this response is a coherent systematic response mediated by an underlying gene regulatory network. Strong similarity between regulatory motifs (**Table 3**) found to be enriched in promoters of stress-responsive genes in *Brachypodium* to those identified in stress experiments in *Arabidopsis* [39], [43] suggests that similar circuits are present in *Brachypodium*. Similar coherency of response was observed for genes related to the cell cycle, as well as conservation of upstream regulatory sequences related to mitosis.

Notable here is the strong co-expression of stress response genes across several experimental conditions. In a recent study of gene expression of barley, many genes responsive to abiotic stress were detected as co-expressed across a large body of expression datasets [52]. This co-expression is impressive, given that expansions in underlying datasets usually causes degradation of co-expression signal (see [53] for an excellent treatment of this phenomenon). That these genes' expression patterns were correlated across more than 1,300 individual arrays is remarkable, and shows the strong regulatory circuitry underlying stress responses in monocot systems.

In addition to this work in barley, in which strong, network-level co-expression of stress-response genes was observed, the response of TF-enriched gene modules to cold stress was also observed in a recent study in rice (*O. sativa* L.; [54]). This response was also observed here, in the population of transcription factors up-regulated in response to cold stress (Module 18, **Table 2** **,** **Figure 3**).

In contrast to the clear coherency of transcriptional regulation of the photosynthetic system, no such coherency was observed for genes related to calcium signaling and binding. Calcium ion binding related loci were sequestered out of modules at a highly significant level (Z-score = −18.1, two-tailed p-value<1e-6), which indicates that unlike more coherently regulated systems, calcium ion binding does not co-express strongly with other genes. Taken in conjunction with the knowledge that calcium-ion binding loci are important for plant abiotic stress response [16], this indicates that the transcript-level expression of these loci simply is not in line with the major modes of plant stress response captured in these experiments. The expression patterns of calcium ion binding loci may differ strongly among tissue types, and the lack of well-defined tissue-specific expression in the current experiment may preclude the resolution of a coherent expression pattern for this group of genes. Dissection of the roles calcium ion binding loci play in abiotic stress response in *Brachypodium* will require further targeted stress experiments.

### Network Plasticity

Analysis of differential correlations for transcription factor/target gene pairs in various conditions revealed a high degree of plasticity in these relationships. The proportion of potentially plastic relationships varied greatly depending on the conditions compared. Neither the conditional comparison with the lowest ratio of potentially plastic gene pair relationships (salt/heat, 241 plastic TF-TG pairs, **Table 4**) nor the comparison with the highest ratio of potentially plastic relationships (salt/cold, 2,027 plastic TF-TG pairs, **Table 4**) were the comparison with the most extreme number of total possible pairings. Of particular interest is the great diversity of differential correlations between salt and drought stresses. There are a large segment of gene pairs that experience very large changes in correlation. More than 11,000 gene pairings had large negative correlations under drought stress and very large positive correlations under salt stress (top right, **Figure 4B**). Conversely, more than 16,000 gene pairings had large positive correlations under drought stress and large negative correlations under salt stress (bottom left, **Figure 4B**). Comparisons between the differential correlations observed between salt and drought stresses and the differential correlations observed between random subsets of the stress data indicate that the differential correlations between salt and drought stresses are unlikely to arise by chance (**Figure 4A**).

The basic underlying assumption of gene co-expression network analysis is that two genes, when co-expressed, can be expected to be reliably co-expressed if there is a biological relationship between them. The stronger the biological relationship between two genes – either due to genuine co-regulation or from necessary co-expression borne of functional relatedness, the higher the correlation in expression between the two genes. The relationships between transcription factor/target gene pairs across conditions are plastic due to dependence on DNA methylation and chromatin modification status, among many other factors. This highlights the importance of inclusion of epigenomic data in any large genomic discovery endeavor.

Because of the possible relationship between TF loci and their target genes, we queried the module membership of the TF loci population, to determine if they were preferentially included or excluded from modules. Similar to the exclusion of calcium ion binding loci from modules, the exclusion of TF loci from modules would indicate that they are more selectively regulated in response to abiotic stress than the loci which are identified to be module members. Of 600 TF loci which are differentially expressed in response to stress, 369 are members of modules. This is significantly fewer TFs than would be expected by chance alone (determined by permutation test, 404.5 loci expected, Z = −3.195, two-tailed p-value = 0.0014). As modules are built on co-expression across many conditions, and it appears the gene co-expression correlations may be plastic, the expectation that TF-TG relationships are consistent across conditions may be incorrect, and the de-enrichment of TFs in modules may reflect that.

In addition to the sequestration of TFs out of modules – which may reflect the plasticity of their relationships to modular genes – genes which are distinctly lacking plastic relationships are of great interest. On the hypothesis that gene co-expression plasticity stems from changes in the underlying biochemical relationship between loci, genes which lack plastic relationships may lack the requisite biochemical changes in regulatory relationships, and may have stable regulatory circuits. Of the 2,752 genes which were considered in the plasticity analysis, 220 genes never showed any plastic relationships to any TF (7.9%). Put another way – the correlation changes across conditions between these genes and the TFs to which they were correlated was always below the significance threshold. Of these 220 genes, 29 were found to have undefined functions. The list of genes which had no plastic relationships also included *Bradi1g42630* annotated as a phosphofructokinase, a loci down-regulated in drought, salt and heat stress, which was a member of module 02. This gene was highly homologous to *AT1G76550*, an *Arabidopsis* phosphofructokinase which functions in primary metabolism and gluconeogenesis. A member of this family in *Arabidopsis* was identified as one of a group of genes which influence plant growth and biomass [55].

A second non-plastic gene is *Bradi5g11640*, which is differentially expressed in response to drought and heat stresses. This gene is highly homologous to *AT1G65960* a glutamate decarboxylase which was found to have its enzymatic activity increase in response to treatment by calcium and calmodulin in combination, indicating that the *Arabidopsis* locus encodes a calmodulin binding protein [56]. The specific role of this locus in *Brachypodium* remains to be elucidated by further molecular experiments.

Sources of gene co-expression plasticity can stem from either the regulator or the target loci. Loci which have particularly stable relationships may represent a group of loci which remain highly accessible to the transcriptional machinery during the four assayed stresses. While this group of 220 genes may be hypothesized to be a ‘core’ group of stress reactive genes, these genes were not enriched for any particular GO term or category.

Based on the dataset used here, we cannot assign cause to the large changes in expression correlation across conditions. It is clear that a full understanding of the abiotic stress response of *Brachypodium* requires epigenomic analysis. With increasing throughput and decreasing costs, full integration of multi-type sequence data waits only on development of novel bioinformatic methods that can take full advantage of rich datasets. The high degree of plasticity observed in the stress response of *Brachypodium* also has implications for whole-genome gene co-expression network reconstruction. Current state-of-the-art software packages, such as WGCNA [26], may be made even more powerful by accounting for the changing relationship between gene pairs across conditions in meta-data enhanced expression datasets. Adopting a ‘regulator-target’ dichotomous view of gene loci – as is common in applications designed for smaller networks – may further improve large network reconstruction efforts.

Weighted gene co-expression analysis of the *Brachypodium* transcriptome under normal growth and four abiotic stress conditions identified 22 modules of genes. Over-expression, knock-down, and knock-out experiments will elucidate the roles of these genes in abiotic stress responses and may guide genetic approaches that confer stress tolerance in economically important grasses. This research provides insight into how this model crop system responds to abiotic stresses. Homology between *Brachypodium* and agricultural target species will allow the identification of stress-responsive target genes in cereal and biofuel feedstock crops, enabling improved stress tolerance in plants critical to serving the needs of society.

We have identified numerous potential transcription factor binding site sequences that are associated with specific expression profiles under abiotic stresses. In addition to correlating these motifs to specific gene expression profiles, we have linked these DNA sequence motifs to specific endogenous plant systems. These candidate *cis*-regulatory sequences may represent key components of the transcriptional circuitries that define the plant's gene regulatory networks. Systems and synthetic biology approaches may take advantage of these circuits to place genes of interest under the control of existing stress response pathways to achieve desirable phenotypes of stress tolerance in agriculturally or economically important crops.

### Web Resources

All microarray datasets are accessible through the *Brachypodium* web genome browser (http://jbrowse.brachypodium.org). The module membership lists, AgriGO GO-enrichment analysis output, and Element promoter content analysis output may be found as supplemental files and are available for download on the Brachypodium.org FTP website (ftp://brachypodium.org/brachypodium.org/Stress/). All individual gene RMA expression stress response profiles for each assayed stress condition may be viewed at the Mockler Lab's plant stress response web portal (http://stress.mocklerlab.org/).

## Methods

### Experimental Growth Conditions and Tissue Sampling

*Brachypodium distachyon* control plants were grown at 22°C with 16 hours light and 8 hours dark in a controlled environment growth room. Abiotic stress conditions included cold, heat, salt, and drought. All treatments were conducted with a light intensity of 200 µmol photons m^−2^s^−1^. For the heat experiments, *Brachypodium* plants were placed in a Conviron PGR 15 growth chamber at 42°C. Cold treatments were conducted in a walk-in cold room maintained at 4°C. Salt stress (soil saturation with 500 mM NaCl) and drought (simulated by removing plants from soil and placing them on paper towels to desiccate) treatments were conducted under the same light and temperature as the control samples. Three-week-old *Brachypodium* plants were placed under the respective conditions two hours after dawn (10 a.m.). Leaves and stems (total above ground tissues) from individual plants were collected at 1, 2, 5, 10, and 24 hours after exposure to the abiotic stress.

### RNA Preparation, Labeled cDNA Synthesis, and Microarray Hybridization

Leaf tissues were pulverized in liquid nitrogen, total cellular RNA was extracted using the RNA Plant reagent (Invitrogen), and RNA was treated with RNase-free DNase essentially as described in [57]. DNase-treated RNA integrity analysis, preparation of labeled target cDNA from *Brachypodium* leaf total RNA, Affymetrix microarray hybridizations, chip scanning, quality control, image processing, and data extraction were performed essentially as described in [58]. One array – heat-stress hour 5 replicate ‘C’ – did not pass quality control and was discarded.

### Mapping of Probes

Probes on the Affymetrix BradiAR1b520742 array were mapped to the Bd21 v1.0 assembly using the Burrows-Wheeler Aligner (BWA) [59]. The Bd21 Brachypodium Array contains 6,503,526 non-control probes. Of these, 99.81% (6,491,341 probes) map to a single location in the genome. Most of the probes (6,491,341) match their target sequences unambiguously with no mismatches in alignment. Only 12,183 probes align with mismatches. All probe sequences represented on the array are entirely distinct from each other. For the probe-set level analysis, probes were associated with annotated genic features. Probes that associated with a single gene's exonic features were collected into strand-specific probe-sets. Only those probe sets associated with the forward strand of a target gene were retained for analysis in differential expression or network prediction. If a probe was associated with exonic features of two genes (if two genes overlap, for instance), that probe was not assigned to any probe set. If a probe was associated with both intronic and exonic features (if a gene has multiple transcripts, or a probe spanned an exon/intron boundary), the probe was not assigned to a probe set. In the 47,960 genic probe sets, each gene was detected by, on average, 31.5 probes. The median number of probes per set was 22.

### Microarray Data Analysis

Probeset level expression values were obtained utilizing the Robust Multi-array Average [25] technique via the Affymetrix Power Tools (APT) software package (http://www.affymetrix.com/partners_programs/programs/developer/tools/powertools.affx). Probe set summarization and expression estimates for each gene were conducted using the apt-probeset-summarize tool (version 1.15.0) from Affymetrix. Data manipulations were performed using Perl scripts. From the resulting signal intensities, differentially expressed genes were calculated using the Significance Analysis of Microarrays (SAM) [60] R package in conjunction with Microsoft Excel.

SAM uses permutations of repeated measurements to estimate the percentage of genes that are identified by chance, representing the false discovery rate. SAM was run with default settings, using 100 permutations, using the ‘two class unpaired’ response type. The S_0_ factor was estimated automatically and no fold-change cutoff was applied at the time of differential expression calling. The Delta value was selected such that the median false-discovery rate was below 0.01. In every case, control and stress RMA expression values were compared in a pairwise fashion within a single stress and time point combination.

### Heatmap and Principal Component Analysis

Heatmap and Principal Component Analysis (PCA) analyses were conducted in R. RMA expression differences between the average expression value per stress time point per treatment were set to saturate at a difference of 4 RMA (such that the maximum value reported in the heatmap was +/− 4 RMA). These expression differences were graphed using the ‘heatmap.2’ function of the gplots package of R. For principal component analysis, the average RMA expression value of each stress time-point, without the above saturation, was used as input for the ‘PCA’ function of the R package ‘factominer’ (http://factominer.free.fr/) [61].

### GO Analysis and Transcription Factor Annotation

Over-represented GO terms were identified using the AgriGO: GO analysis toolkit (http://bioinfo.cau.edu.cn/agriGO/) [27]. Analysis was done by comparing the number of GO terms in the test sample to the number of GO terms within a background reference. Over-represented GO terms had a FDR corrected *P*-value of less than 0.05 and more than 5 mapping entries with a particular GO term. GO-terms were assigned to genes based first on InterProScan [35] results for the entire predicted proteome of the *Brachypodium distachyon* MIPS version 1.2 annotation [24]. Approximately 40% of genes did not have any GO-terms associated with them. Gene products from this set that had high-quality BLASTP matches to *Arabidopsis thaliana* gene products were assigned the same set of GO terms that their *Arabidopsis* homolog possessed. The list of putative *Brachypodium* transcription factors was obtained from gene annotation queries and BLASTP comparisons to rice (*Oryza sativa*) transcription factors, which were obtained from Plant Transcription Factor Database (http://plntfdb.bio.uni-potsdam.de/v3.0/) [62].

### Network Analysis

Normalized RMA expression values for 9,496 differentially expressed genes were loaded into the R package WGCNA [26]. An adjacency matrix was calculated using B = 23. Distance metrics between profiles were calculated using the TOMdist function using an un-signed TOM type. Hierarchical tree solution was calculated using the flashClust [63] function with the ‘method’ option set to ‘average’. Modules were called using the moduleNumber function, cutHeight = 0.91, and minimum module size was set to 25. Module colors were set using labels2colors. These modules were merged, using mergeCloseModules, a cut height of 0.1, iteration set to ‘true’, and enabling re-labeling. Final module colors and numbers were set as a result of this merging. Modules were exported for visualization in Cytoscape [64] using the “exportNetworkToCytoscape” function in the WGCNA R package and an adjacency threshold of 0.35. Once imported to Cytoscape, edges were filtered for a minimum value of 0.45, and the final network layout was obtained using the “Force Directed” in-built Cytoscape layout method. Cytoscape-layout and edge filtering caused some modules to not be connected by edges. These were not included in final Cytoscape layout; however, their mutual connectivities in the adjacency matrix served to allow WGCNA to call them as modules so they were analyzed as such for AgriGO-mediated GO enrichment and for Element-mediated promoter analysis. Only those modules that were graphed in Cytoscape as being interconnected with edges above the 0.45 cutoff were included in the final figures.

### Promoter Analysis

Genes were grouped based on module membership. Based on the MIPS version 1.2 *Brachypodium distachyon* annotation, the 500 nucleotides directly upstream of each gene was extracted from the *Brachypodium* genome. The promoters for the genes in each module were analyzed on a module-by-module basis using Element [28]. The set of all predicted promoters in the genome were analyzed using the ‘bground’ command using all possible 5 to 8 nucleotide sequences as the set for analysis. This formed the set of background motif occurrence statistics against which module groupings of promoters were compared. Motif occurrences in module sets of genes were then compared against the background set. Motifs shorter than 5 nucleotides in length are expected to fall into one of two categories – background false-discoveries or true-positives that will be contained within larger, also significant motifs. Transcription factor binding sites longer than 8 nucleotides in length are expected to either overlap or be multi-partite motifs, both of which will generate significant sub-motifs in this analysis. In some cases, for specific examples, membership lists from two modules were combined for analysis by Element. Element was run using default cutoffs for significance (FDR<0.01), on 16 processors (‘-t 16’).

### Network Plasticity Analysis

Network plasticity was determined by comparing the correlation of gene pairs between conditions. Between two conditions, every gene that was called by SAM as being differentially expressed in both conditions was segregated into one of two groups – the TF group or the non-TF group. Putative *Brachypodium* transcription factors were identified as described above. All pairwise Pearson's correlation values were calculated between groups in each of the conditions. This yielded two correlation values for each gene pair – one value corresponding to each condition. The order of the values of each gene expression profile across all assayed stress conditions was then randomly shuffled via the Fisher-Yates Shuffle procedure [65] creating 7,200 random permutations of the data. In each permutation, two subsets of equal size (N = 15) were selected. Each permutation therefore was a random permutation of a gene's total expression data profile from which two independent samples of size N = 15 were selected. The pairwise Pearson's correlations between all TF-TG pairs were calculated in each permutation. In order to determine significance of correlation change across conditions, a cutoff was chosen such that the average number of gene pairings that had correlation changes exceeding that cutoff in each random permutation (average number of false discoveries per permutation) was an appropriately small ratio of the number of gene pairs that had correlation changes exceeding that threshold in the true dataset (number of positives). This process is similar to SAM [60]. In all comparisons, the threshold was chosen such that the FDR was less than or equal to 0.05.

### Undefined Module Member Genes

In order to identify genes which could be associated with a role in abiotic stress response by module membership, but could not have a predicted function attached to them, the entirety of the *Brachypodium* proteome was aligned against the Phytozome annotations for *Sorghum bicolor* [66], *Glycine max* [67], *Arabidopsis thaliana* [68], *Zea mays* [69], *Setaria italica* [70], and *Oryza sativa* [71]. Proteins, which aligned with 70% identity over 70% or more of their total length, to a gene in one of the target species, were associated with the functional annotation of the target gene. Of 26,552 *Brachypodium* proteins, 15,480 (58.3%) aligned to at least one target gene in at least one target species. 11,072 genes (41.7%) did not align to any target genes in any target species. Of those genes that aligned, 1,313 were associated only with annotations such as “expressed”, “putative protein”, “protein of unknown function”, or similar, and never with more functionally-informative annotations. These were identified as undefined loci. In order to supplement these associations, InterProScan [35] annotations were included. Genes which did not have an informative InterProScan result, and did not align to a target species, or, did not have an informative annotation if they did align, were identified as undefined gene loci. Therefore, the only information we could reliably attach to these loci were their expression profile and the set of genes with which they co-express.

### Accession Number

The raw data is available at the Plant Expression Database (www.plexdb.org) under PLEXDB accession number ‘BD2’.

## Supporting Information

Figure S1 Principal component analysis of RMA normalized microarrays.
(PDF)

Figure S2 Heatmap of RMA-expression value differences for 359 calcium ion binding associated loci.
(PDF)

Figure S3 Principal component analysis of RMA normalized microarray data for 359 calcium ion binding associated loci.
(PDF)

File S1 Gene membership lists for all 22 modules.
(XLS)

File S2 AgriGO output for all 22 module gene lists.
(XLS)

File S3 Element output for all 22 module gene lists.
(XLS)
