# Supplementary figures and images for: Analysis of Global Gene Expression in Brachypodium distachyon Reveals Extensive Network Plasticity in Response to Abiotic Stress
Source: PLoS One. 2014 Jan 29;9(1):e87499. doi: 10.1371/journal.pone.0087499 (PMC3906199; doi:10.1371/journal.pone.0087499)

**Supplemental  
Figure S1.**  
Principal  
component  
analysis of RMA  
normalized  
microarrays.

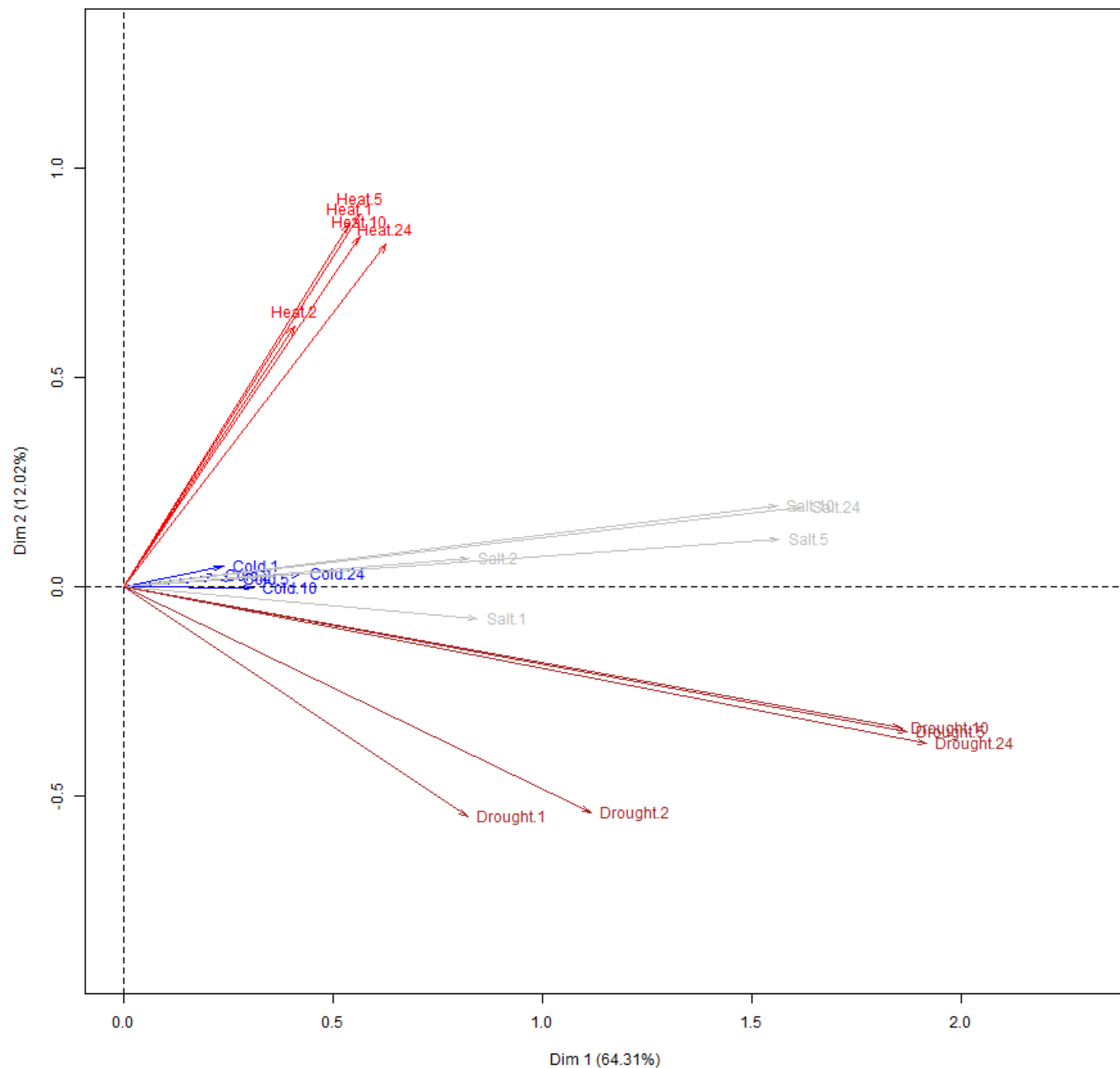

Supplement: Figure S1 — Principal component analysis of RMA normalized microarrays. (PDF) [file pone.0087499.s001.pdf]
