# Supplementary material for: Analysis of Global Gene Expression in Brachypodium distachyon Reveals Extensive Network Plasticity in Response to Abiotic Stress
Source: PLoS One. 2014 Jan 29;9(1):e87499. doi: 10.1371/journal.pone.0087499 (PMC3906199; doi:10.1371/journal.pone.0087499)
Supplement: Figure S2 — Heatmap of RMA-expression value differences for 359 calcium ion binding associated loci. (PDF) [file pone.0087499.s002.pdf]

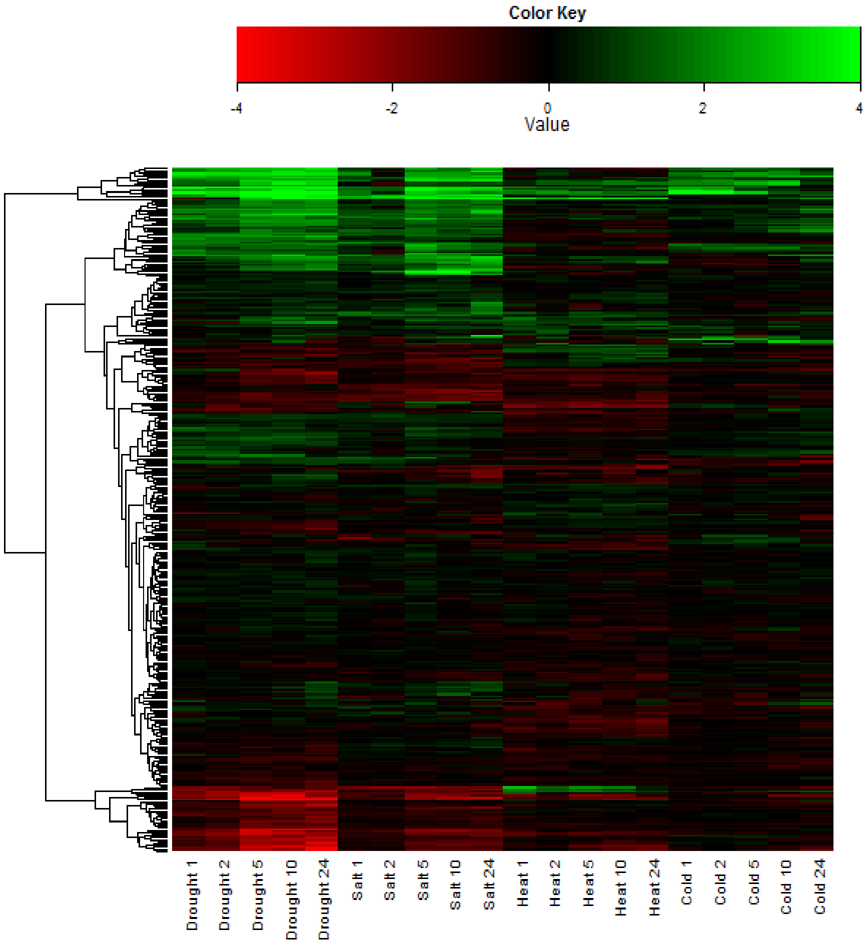

Supplemental Figure S2. Heatmap of RMA-expression value differences for 359 calcium ion binding associated loci.
