# Supplementary material for: Analysis of Global Gene Expression in Brachypodium distachyon Reveals Extensive Network Plasticity in Response to Abiotic Stress
Source: PLoS One. 2014 Jan 29;9(1):e87499. doi: 10.1371/journal.pone.0087499 (PMC3906199; doi:10.1371/journal.pone.0087499)
Supplement: Figure S3 — Principal component analysis of RMA normalized microarray data for 359 calcium ion binding associated loci. (PDF) [file pone.0087499.s003.pdf]

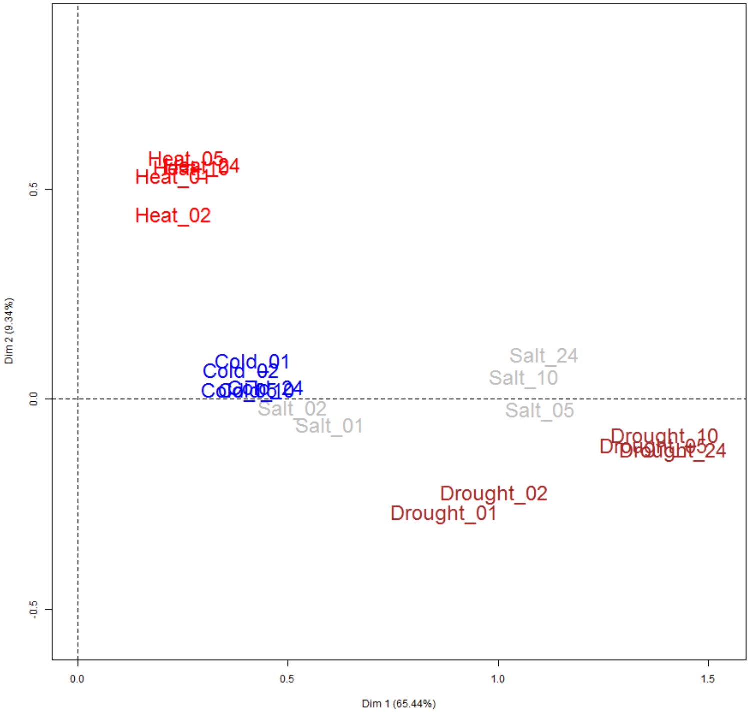

Supplemental Figure S3. Principal component analysis of RMA normalized microarray data for 359 calcium ion binding associated loci.
